# Biosignals, facial expressions, and speech as measures of workplace stress: Workstress3d dataset

**DOI:** 10.1016/j.dib.2024.110303

**Published:** 2024-03-08

**Authors:** Gulin Dogan, Fatma Patlar Akbulut, Cagatay Catal

**Affiliations:** a Department of Computer Engineering, Istanbul Aydın University, Istanbul, Turkey; b Department of Software Engineering, Istanbul Kültür University, Istanbul, Turkey; c Department of Computer Science and Engineering, Qatar University, Doha, Qatar

**Keywords:** Physiological signals, Face expression, Audio signal, Survey Stress, Wearable device

## Abstract

WorkStress3D is a comprehensive collection of multimodal data for the research of stress in the workplace. This dataset contains biosignals, facial expressions, and speech signals, making it an invaluable resource for stress analysis and related studies. The ecological validity of the dataset was ensured by the fact that the data were collected in actual workplace environments. The biosignal data contains measurements of electrodermal activity, blood volume pressure, and cutaneous temperature, among others. High-resolution video recordings were used to capture facial expressions, allowing for a comprehensive analysis of facial cues associated with tension. In order to capture vocal characteristics indicative of tension, speech signals were recorded. The dataset contains samples from both stress-free and stressful work situations, providing a proportionate representation of various stress levels. The dataset is accompanied by extensive metadata and annotations, which facilitate in-depth analysis and interpretation. WorkStress3D is a valuable resource for developing and evaluating stress detection models, examining the impact of work environments on stress levels, and exploring the potential of multimodal data fusion for stress analysis.

Specifications Table

**Table:** 

Subject	Data science Medical science
Specific subject area	Signal processing, Stress classification
Type of data	Physiological Signals, Facial Expressions, Sound Signals
How the data were acquired	Participants were provided with a series of three-part questionnaires. The first section was devoted to personal inquiries, the second section utilized the PANAS Scale to measure emotions, and the third section consisted of questions designed to assess overall stress levels. During this stage, participants answered a total of 36 questions. After completing the ques- tionnaires, participants were required to document random physiological data and complete six-question instant assessment questionnaires. The physiological signals were gathered using the wearable device Empatica E4. In addition, participants were asked to provide facial approximations and voice data at specific times during the study.
Data format	Facial expressions: pixel Audio signals: raw Physiological signals: time series Questionnaire: raw
Data collection	In our study, we collected the experiences and emotions of participants using the experience sampling (ES) method. The ES was conceived as a half-time-based approach, occurring semi-randomly between 10 a.m. and 8 p.m., with seven notifications per day incorporated into participants' daily routines. Six times per day, participants' devices prompted them to complete instant surveys. During these 15-minute periods, the participants' electrodermal activity, blood pressure changes, and cutaneous temperature were recorded. Moreover, when participants encountered intense emotions throughout the day, they were randomly reminded to take personal photographs and audio recordings in order to share their experiences. It is essential to observe that none of our research methods involved the deliberate induction of stress. Instead, the emphasis was placed on identifying and evaluating the tension that routine activities impose on individuals.
Data source location	Institution: Istanbul Kultur University, Department of Computer Engineering City/Town/Region: Istanbul, Bakırköy, Ataköy Country: Turkey Latitude and longitude (and GPS coordinates, if possible) for collected samples/data: 41° 5 7.3284’’ N 29° 2 22.3836 ’ E
Data accessibility	Repository name: Mendeley Data Data identification number: 10.17632/t93xcwm75r.10 Direct URL to data: https://data.mendeley.com/datasets/t93xcwm75r/10

## 1 Value of the Data

The incorporation of multiple modalities, including speech signals, biosignals (such as electrodermal activity, blood volume pressure, and skin temperature), and facial expressions, establishes a complete and multi-faceted method for comprehending workplace stress. The presence of this diversity matters because it enables a comprehensive analysis of stress indicators, encompassing both physiological and behavioral components linked to stress. For example, biosignals provide information about the body's reaction to stress, facial expressions give subtle emotional signals, and speech signals reflect tension through words. The benefit resides in the synergy of different modalities, which offers a more intricate comprehension of stress dynamics than any individual modality could offer. This dataset is diverse and allows for a more thorough investigation into the ways in which different stressors appear in different aspects. It enhances stress analysis and detection methods by including a wider range of indicators connected to stress. The WorkStress3D dataset offers several key benefits, which can be summarized in the following concise key points:

• The integration of speech, biosignals, and facial expressions allows for a thorough examination of workplace stress, offering a comprehensive understanding of both the physiological and behavioral aspects of stress responses.
• The dataset is gathered from real office settings, ensuring that it accurately represents stress events and can be practically applied to learn how stress works in professional environments.
• The dataset provides a comprehensive depiction of stress levels, covering both stress-free and high-stress work contexts. This allows for a detailed examination of stress manifestations across a wide range of scenarios.
• The dataset is accompanied by detailed metadata and annotations, which enable researchers to perform thorough analysis, create advanced stress detection algorithms, and explore innovative uses of multimodal data fusion for comprehensive stress assessment.
• The rich multimodal data in the WorkStress3D dataset enables the identification of patterns, extraction of features, and building of machine learning models for the categorization and analysis of stress. This dataset enables researchers to identify correlations between speech, biosignals, and facial expressions in order to detect indicators of stress. The study of stress triggers and manifestations is applied to real-life professional situations, aiding in the understanding of how individuals respond to stress. By incorporating examples from both stress-free and high-stress contexts, a dataset is utilized to authenticate and contrast stress signs, thereby establishing reliable indicators for stress classification.

## 2 Data Description

**Fig. 1:**
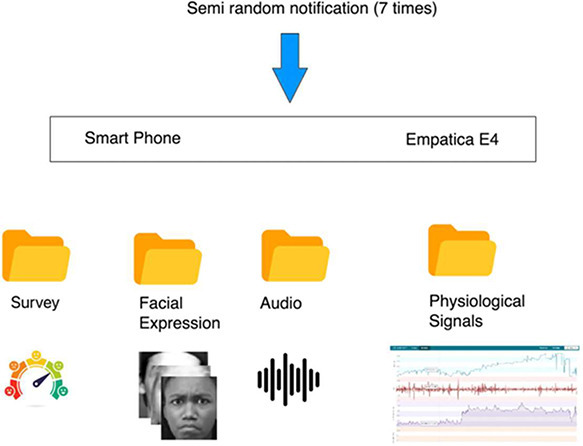
The data collection protocol and folder structure.

It is quite tough to solve the problem of stress in today's modern culture [1]. According to Lazarus [2] and Frankenhaeuser [3] many aspects of an individual's life can expose them to a wide range of stresses, each of which can come in varying degrees of intensity. People are said to suffer stress when they deviate from their usual routines and step outside of their comfort zones. This type of behavior is considered to be the cause of stress. Individuals are rendered unable to properly handle risks to their physical, emotional, or psychological well-being as a result, and it has the potential to lead to chronic disorders [4]. The purpose of the research is to make a contribution to this area of study by developing a dataset that is capable of identifying signs of stress. This data collection consists of questionnaires as well as physiological data, and its purpose is to explore the influence that stress has on human physiology. The dataset contain four main components: survey data, biosignals, facial expressions, and audio signals. Fig. 1 displays the visuals that illustrate the data collection protocol and folder structure.

The primary component of the dataset consists of a wide range of survey responses, including demographic information, immediate mood assessments, evaluations using the PANAS scale, and a comprehensive assessment of general stress. The demographic survey was used to obtain general information about the current group of participants. These survey questions are described in more detail in Table 1. The present mood of the participants is assessed through the use of instant survey questions displayed in Table 2, which are administered via an experience sampling application. This survey is designed to be completed multiple times by participants over a period of 7 days, at various times throughout the day.

**Table 1:** Description of demographic survey.

Data	Data Type	Data Description
Participants	Integer	Participant's id number
Gender	String	Participant's age
Age	Integer	Participant's gender
Height	Integer	Participant's height (cm)
Weight	Integer	Participant's working type (public or private or not working)
Industry	String	Participant's marriage status
Occupation	String	Participant's profession
Marital Status	Boolean	Whether the participant is married or not
Smoking Status	Boolean	Whether the participant's smoke or not
Chronic Disease	Boolean	Whether participant's has disease
Drug Status	Boolean	Whether participant's use medication

**Table 2:** Instant survey questions of the experience sampling study.

Question	Options
How do you currently feel?	Happy, Unhappy, Good, Bad, Neutral, Relaxed, Satisfied, Energetic, Excited, Tired, Nervous, Sad, Angry, Worried, Lonely, Guilty, Sick, and Other
What are you doing at the moment?	Work, rest, food/drink, cleaning, sports, hobby, awareness work, listening to music, watching videos, free time, and other
Is anyone else with you?	Manager, Owner, Colleague, Family Members, Partner, Friend, Strangers, Pets, and Nobody
Is there someone or something currently troubling you?	Yes, No and I Don't Know
Would you prefer to be somewhere else right now?	Yes, No and I Don't Know
Do you have the energy to complete additional tasks today?	Yes, No and I Don't Know

The general stress test calculates a stress score by analyzing participant responses to twenty scenarios reflecting everyday life situations (Table 3). This assessment is influenced by significant situations, such as making determinations about crossing a street when the traffic light is on the verge of changing [6].

**Table 3:** General stress survey.

Question no	Question
1	Are you attempting to juggle multiple jobs in a short period of time?
2	Do you grow impatient in cases of business disruptions or delays?
3	Do you always feel like you have to win in the games you play, even if it's for fun?
4	Do you try to cross the traffic light with your car when the red light is about to turn on?
5	Even if you need help with something you do, would you refrain from asking?
6	Do you always feel the need to earn the admiration of others and be respected?
7	Do you always criticize the way others do their job?
8	Do you frequently look at your watch or a clock?
9	Do you ever have excessive ambitions to improve your achievements and position?
10	Do you get the idea that time is not enough for you?
11	Do you have the habit of doing more than one task at once?
12	Do you often feel nervous or angry?
13	Do you find it difficult to find time for yourself and your hobbies?
14	Do you have a tendency to talk quickly or speed up conversations?
15	Do you consider yourself a difficult person to get along with?
16	Do your friends or relatives say that it's hard to get along with you?
17	Do you have a tendency to be involved in more than one project?
18	Do you often set deadlines for finishing your work?
19	Do you feel guilty when you take time off to rest or sit idle?
20	Do you ever put too much responsibility on yourself?

The final survey is the short PANAS scale [5] is used to measure both positive and negative emotions. These survey questions are shown in Table 4.

**Table 4:** Short PANAS.

Question no	Question
1	Active
2	Attention
3	Vigilance
4	Angry
5	Hostility
6	Determined
7	Inspired
8	Fear
9	Unhappiness
10	Shame

The physiological signal file comprises data from Empatica E4 wristband sensors, including electrodermal activity (EDA), blood volume pressure (BVP), skin temperature, and accelerometer (ACC). The EDA, which includes skin conductivity, phasic, and tonic components, is sampled at a rate of 4 Hz. A discriminative threshold of around 0.05 milliseconds is utilized in the skin conductivity reading to differentiate between tonic and phasic characteristics. Consistently, skin temperature is measured at 4 hertz and denoted in degrees Celsius. Blood pressure information is obtained by sampling the Blood Volume Pulse (BVP) signal at a constant rate of 64 hertz (Hz) on a consistent basis. Continuously collected at 64 Hz are accelerometer readings that quantify gravitational force (g) in three spatial dimensions (x, y, and z). A meticulous examination of physiological responses and continuous monitoring of activity levels are guarantees by this comprehensive approach. In order to mitigate the impact of signal frequency variability, a prudent downsampling technique was applied to standardize the signals. By selecting a downsampling frequency of 4 Hz, a harmonized and efficiently fused dataset can be obtained, which represents an ideal compromise between the preservation of information and computational efficiency. The additional component comprises facial expressions that are categorized as either non-stressful (0) or stressful (1) based on the level of stress indicated by numerical values. In order to guarantee a precise portrayal of emotional states, the facial expression data is encoded as pixels within grayscale images measuring 48×48. In both stressful and non-stressful situations, the pixel-based representation enables nuanced interpretation and analysis of facial expressions. The ultimate element, labelled “Audio,” comprises unprocessed audio data that was collected directly from participants. By providing this audio component, which contributes to a comprehensive understanding of the various manifestations of stress in the dataset, it serves as a foundational element for subsequent auditory analysis.

## 3 Experimental Design, Materials and Methods

### 3.1 Study protocol

**Fig. 2:**
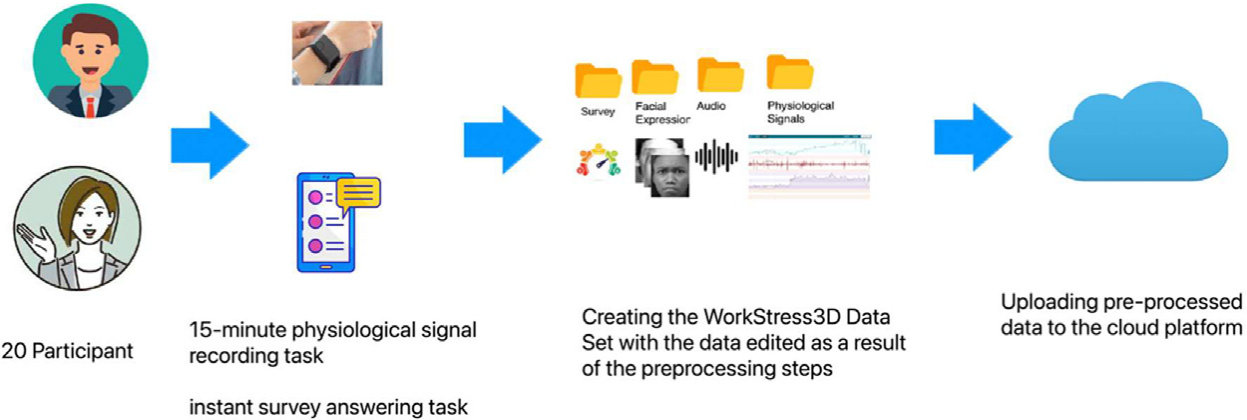
Stages of preparation of the WorkStress3D dataset.

The ES technique was meticulously designed as a semi-random schedule, wherein participants were notified between 10 a.m. and 8 p.m. on a daily basis for seven days. The participants were prompted by these notifications to engage in a range of activities, including completing instant surveys, simultaneously recording physiological signals, and providing voice and facial expression data. The duration of the physiological signal recording, which was synchronized with the instant survey task, was fifteen minutes. The participants engaged in an active process of documenting their emotional experiences by means of capturing photographs and creating audio recordings. By employing this methodology, an extensive and reliable data collection process was established, as participants contributed six sets of data daily for the duration of the seven-day study. The study sample comprised a total of twenty individuals, of which around 35% were female and 65% were male, with an average age of 38.7 years. Significantly, the research avoided the implementation of simulated stress conditions, preferring instead to evaluate stress levels in the genuine environment of routine workplace settings. The investigation of workplace stress was conducted methodically across a wide range of professions, guaranteeing the collection of a representative cross-section of stress experiences in society. The purposeful incorporation of occupations characterized by both high and low levels of stress enabled a comprehensive examination, thereby enhancing our understanding of stress in all its diverse forms. The experience sampling method utilized in the study is depicted in Fig. 2, which provides a systematic outline of the approach to stress assessment within the research framework.

### 3.2 Study population

**Fig. 3:**
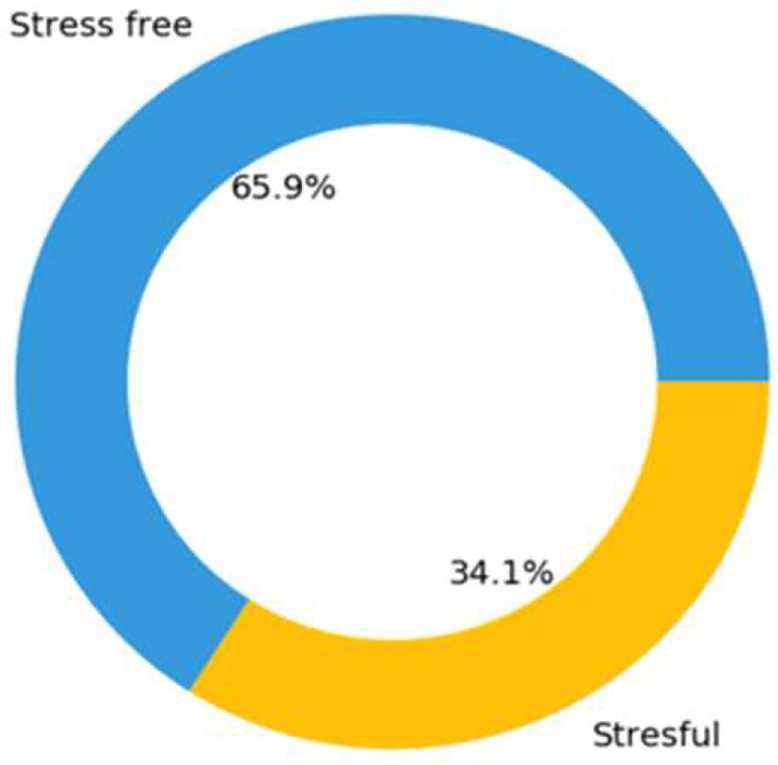
Stress distribution of data.

The population of the study consisted of a varied collection of 20 subjects, from whom a total of 4,200 responses to questionnaires and 175 hours of physiological signals (equal to 168,000 samples) were gathered. There were 35% women and 65% men among the participants, and the average age was 38.7 years old. A marketer, an entrepreneur, an academic, a secretary, and an individual who was self-employed were among the people who took part in the study. Additionally, there were five computer engineers, four research assistants, two judges, two lawyers, two doctors, two self-employed individuals, and two doctors. Seventy percent of the participants were working jobs in the private sector, and the remaining thirty percent had positions in the public sector. When the habits of the participants were investigated, it was discovered that 75% of them did not smoke, while the remaining 25% admitted to being smokers. In terms of the conditions affecting their health, twenty percent of the participants admitted to suffering from some kind of ailment, while eighty percent of them considered themselves to be in good health. The participants who reported having diseases comprised two people who had eczema, one person who had a history of cardiac problems, and one person who had a less serious medical condition. The study population consisted of individuals from a wide range of demographic groups and professional backgrounds. This allowed for a thorough representation of persons working in a variety of jobs and industries, which increased the generalizability of the findings to settings that are more representative of the real world. Finally, the stress distributions of the data contained in this data set are shown in Fig. 3.

### 3.3 Facial expression and audio data

The photographs of facial expressions were taken with smartphones, and they were initially captured at a resolution of 1152×2048 pixels in RGB format. It was essential to separate the facial expressions from the background in order to make the process of feature extraction and image processing more manageable. This was achieved by converting the images to binary format, which is a format that is frequently utilized in activities involving pattern recognition. In addition, in order to make the most efficient use of the computer's resources, each photograph was scaled down to the lowest possible resolution that could be used in the research. After that, the dimensions of the facial expressions were fixed at 48 by 48 pixels, and they were grayscale. The dataset contains a total of 83 different examples of facial expressions, each of which is characterized by the aforementioned criteria. These facial expressions reflect the participant's immediate emotion. Momentary emotions are categorized according to whether the person is stressed or not. In terms of the audio data, the participants shared their recordings in the raw format in which they were originally recorded. For the purposes of this study, a total of 68 audio recordings were gathered.

### 3.4 Measurements of physiological signals: Empatica E4

The formation of this dataset aimed at conducting a thorough analysis of stress, incorporating various physiological signals. Acknowledging the high costs associated with creating a diverse dataset, efforts were made to optimize cost-effectiveness while maximizing the number of observations. In pursuit of this goal, the selection of devices played a pivotal role, prioritizing ease of use, applicability, and coverage of targeted data. Consequently, the study chose to leverage the practicality of a wearable device and a smartphone. A hybrid approach was adopted to examine the impact of stress on human physiology. The Empatica E4 smart bracelet was employed to capture signals measuring individuals' physiological data. Notably, the Empatica E4 is a wearable health technology device capable of measuring various biological parameters. The device provided signals encompassing blood volume pressure (BVP), electrodermal activity (EDA), body temperature (TEMP), and 3-axis accelerometer (ACC), as outlined in Table 2. The Empatica E4 effortlessly stores these signals, facilitating convenient storage in the cloud through a mobile software application named E4 Realtime. The signals obtained from this device are blood volume pressure-BVP, electrodermal activity-EDA, heart rate-HR, body temperature-TEMP, 3-axis accelerometer-ACC signals, as given in Table 5.

**Table 5:** Empatica E4 data description.

Data	Sample Rate	Description
BVP	64Hz	This signal gauges heart rate, enabling the detection of variations that occur when stress or tension levels are elevated.
EDA	4Hz	This signal data is employed to quantify the electrical activity of the skin.
ACC	32Hz	This signal data is utilized for assessing movement and activity levels. With this information, one can measure an individual's activity level, count steps, and determine their mobility status.
TEMP	4Hz	This signal data is employed for measuring the skin's surface temperature. It is utilized to monitor the individual's stress levels, exercise, and overall health status.

The Empatica E4 wristband was used to carry out the continuous collection of physiological signals. Due to the fact that these signals were sampled at various frequencies, there may be inconsistencies in their temporal resolution. In order to solve this problem, a subsampling method was utilized so that a more accurate representation of the signal frequencies could be obtained. The gathered physiological data were then divided into three distinct window sizes of 15, 30, and 60 seconds respectively so that further analysis could be performed. This segmentation made it possible to conduct a more in-depth analysis of the signals within the designated time intervals.

### 3.5 The questionnaire data

The study sought to acquire unbiased and objective data from participants, as any biases could lead to erroneous conclusions. To accomplish this, careful consideration was given to the design and order of the survey questions, as responses to earlier questions may affect those to later questions. The inclusion of open-ended questions allowed participants to express their thoughts in their own terms, while Likert-type questions provided a variety of response options [7]. While open-ended inquiries provide more information, they may also result in a greater number of data gaps [8]. To address this, we utilized Likert-type scale surveys with 4- and 5-point rating scales, which are widely recognized as effective scoring methods in the literature. The study employed a participant demographic information questionnaire consisting of eleven questions to obtain insight into each participant's general profile. Questions included birth date, age, gender, height, weight, marital status, occupation, employment sector, smoking patterns, disease status, and medication use, if applicable. In addition, an instant survey task was completed simultaneously with the collection of physiological signals. Participants were specifically instructed to choose one response for each of the first three “instant” questions in the questionnaire. These questions have been carefully prepared by us in order to understand the instant emotional state and have been addressed in a way that each question has an answer.

In addition to the questionnaires previously mentioned, the study employed two additional surveys: a 20-item general stress test [6] and a 10-item short PANAS scale [5]. The general stress test uses a four-point Likert- type scale to evaluate individuals’ responses to 20 daily events and calculates a general stress score based on the results. General stress testing from Sweet Briar University Academic Resource Center; Responses were rated on a 4-point Likert- type scale ranging from 0 (very little) to 4 (very much). For example, participants are asked, “Would you attempt to cross the street in your car as the traffic light is about to turn red?” and each response is assigned a numeric value. The total score is then classified into different stress levels: 20 to 30 indicates a need to improve coping strategies; 31 to 50 indicates effective stress management; 51 to 60 indicates an elevated stress level, bordering on extreme anxiety; and 61 or higher indicates a high risk of developing heart disease. If the result of this test was below 60 points, we considered the stress level as normal, and if it was above 60 points, we considered the stress level as high. On the other hand, the short PANAS scale requires participants to evaluate their emotional states using a five-point Likert-type scale, which includes five positive and five negative emotions. When evaluating the PANAS scale, responses are rated on a 5-point Likert-type scale ranging from 1 (very little or not at all) to 5 (very much).

The purpose of collecting these survey data was to make statistical inferences about the participants’ behaviors and emotional states [9], [10], [11], [12]. Using a four-point Likert-type scale, the general stress test provides feedback by assessing individuals’ stress levels in response to ordinary life events. In this way, the general stress test, the short PANAS scale, and instantaneous surveys are incorporated into the comprehensive data collection procedure of the study. These instruments serve to better comprehend and analyze the emotional experiences of study participants, thereby facilitating the investigation of stress patterns and coping mechanisms within the study population.

### 3.6 Data preprocessing

The information that was gathered is put through an essential preprocessing stage, which incorporates both image and physiological data. Regarding images of facial expressions, they are first obtained at a resolution of 1152 by 2048 pixels and displayed in RGB colors. It is essential to separate the actual facial emotion from the background in order to make feature extraction and image processing easier. Because of this, the images are transformed into the binary format [13]. In addition, in order to make the image resolutions consistent across the board, each image is shrunk down to the lowest resolution that was considered for the study. When it comes to physiological signals, they can be gathered at frequencies ranging from 4 Hz to 32 Hz and even 64 Hz, depending on the sensors that are being utilized. A downsampling technique is utilized so that all physiological signals can be brought to a frequency that is constant at 4 Hz. This helps to streamline the analysis and ensures that it is uniform. Because it helps find a compromise between computing economy and accuracy, downsampling is a useful technique in management that focuses on cost-performance analysis [14]. After that, quadratic feature transformations are applied to the equalized physiological signals, and polynomial feature transformations are used to supplement the dataset. The data are further enriched by this process, which also contributes to the later analysis's ability to differentiate between distinct groups.

## CRediT authorship contribution statement

**Gulin Dogan:** Data curation, Visualization, Investigation, Writing – original draft, Writing – review & editing. **Fatma Patlar Akbulut:** Supervision, Conceptualization, Methodology, Investigation, Writing – review & editing. **Cagatay Catal:** Investigation, Writing – review & editing.

## Data Availability

WorkStress3D (Original data) (Mendeley Data).
